# Novel polymorphisms within the *Dlk1-Dio3* imprinted locus in rat: a putative genetic basis for strain-specific allelic gene expression

**DOI:** 10.3389/fgene.2012.00296

**Published:** 2012-12-14

**Authors:** Laura J. Sittig, Eva E. Redei

**Affiliations:** Department of Psychiatry and Behavioral Sciences, The Asher Center, Feinberg School of Medicine, Northwestern UniversityChicago, IL, USA

**Keywords:** hippocampus, iodothyronine deiodinase-III, allelic expression, imprinting, parent-of-origin, rat, polymorphism

## Abstract

The imprinted iodothyronine deiodinase-III (*Dio3*) thyroid hormone metabolizing gene exhibits paternal expression in most fetal tissues, yet exhibits aberrant, maternal expression in the hippocampus in F1 offspring of Sprague Dawley (SD) × Brown Norway (BN) rats. The maternal hippocampal expression is associated with lower *Dio3* mRNA levels specifically in the hippocampus. Here, we tested the hypothesis that genetic polymorphisms between the SD and BN parent strains cause this aberrant allelic *Dio3* expression and contribute to behavioral sequelae of higher thyroid hormone levels locally in the hippocampus, including anxiety-related behavior. We mapped and sequenced the *Dio3* gene and several previously unmapped regions in the *Dlk1-Dio3* locus that could regulate imprinting of the *Dio3* gene. In the *Dio3* promoter we identified four novel polymorphisms between the BN and SD strains. Next we took advantage of the fact that the Long Evans (LE) strain exhibits identical polymorphisms as the SD strain in the region 5' and including the *Dio3* gene. By reciprocally crossing LE and BN strains we tested the relationship among *Dio3* promoter region polymorphisms and *Dio3* mRNA expression in the hippocampus. Aberrant strain-specific hippocampal *Dio3* allelic expression replicated in the LE-BN reciprocal crosses, suggesting that hippocampal-specific imprinting of the *Dio3* gene is not the result of a unique genetic or epigenetic characteristic of the SD rat strain, or a unique epistatic interaction between SD and BN. To our knowledge no other studies have reported a genetic × epigenetic interaction of genetic origin in the brain.

## Introduction

Parent-of-origin allele-specific gene expression, often called genetic imprinting in its complete form, is enriched in the brain. In fact, widespread brain region-specific allelic expression has been reported (Gregg et al., 2010) [but see Deveale et al. (2012)], and has been hypothesized to be related to the different functions served by different regions of the brain. Examples of neurodevelopmental disorders resulting from genetic mutations in imprinted genes or imprinting regulatory regions include Prader-Willi, Angelman, and Rett syndromes (Davies et al., 2005; Kernohan and Berube, 2010), yet it is not known how or if brain region-specific allelic expression plays into these disorders. Apart from overt syndromes, it is possible that genetic differences in imprinted genes or imprinting regulatory regions that do not necessarily cause disease could differently affect the function of specific brain regions and behavioral vulnerability. Defining and understanding such interactions will be increasingly important toward understanding the normal brain and neuropsychiatric disorders.

In recent studies, we identified one example of brain region-specific, parent-of-origin specific allelic expression of the gene encoding the thyroid hormone metabolizing enzyme, *Dio3*. We crossed two rat strains [Sprague Dawley (SD), S and Brown Norway (BN), B] to produce reciprocal crosses, abbreviated SB and BS with maternal strain indicated first. The *Dio3* gene showed preferential paternal expression in the fetal brain and biallelic expression in adult brain of the BS offspring similarly to the expression profile reported previously (Edwards et al., 2008; Gregg et al., 2010). However, the SB offspring showed a preferential maternal *Dio3* expression in hippocampus that was present in both fetal and adult brain, and corresponded to lower total expression of *Dio3* mRNA. The resulting sequelae included a higher level of T3 in the hippocampus (as the Dio3 enzyme is responsible for inactivating thyroid hormones) and several anxiety-related behaviors (Sittig et al., 2011a). The relationship between hippocampal *Dio3* expression, lower total *Dio3*, elevated T3 in hippocampus, and anxiety-related deficits was substantiated in a separate study using SB-BS reciprocal crosses, in which the SB difference in hippocampal *Dio3* expression was exacerbated by exposure to prenatal alcohol, producing further elevation in hippocampal T3 levels and behavioral deficits (Sittig et al., 2011b). Here, we aim to confirm a genetic cause for aberrant hippocampal *Dio3* allelic expression, show candidate causative polymorphisms, and test their effect *in vivo* using a novel reciprocal rat cross.

The *Dlk1-Dio3* locus harbors a set of developmentally regulated genes that show conservation across vertebrates (Edwards et al., 2008). Eutherian mammals including human, rat, and mouse imprint this locus, where the paternally expressed genes are *Dlk1, Rtl1*, and *Dio3*, and the maternally expressed genes include non-coding RNAs (ncRNAs), small nucleolar RNAs (snoRNAs) in the rat, and the *Gtl2* gene (Royo et al., 2007). Here, we sequenced SD and BN DNA at regions that could potentially regulate *Dio3* imprinting. The first candidate region is the *Dio3* promoter region, if a hippocampal-specific transcription factor interacts with a polymorphic site. Another region that could influence hippocampal *Dio3* allelic expression patterns is the intergenic differentially methylated region (IGDMR) located between *Dlk1* and *Rtl1.* This region has a series of CpG sites that are methylated on the paternal allele but unmethylated on the maternal allele. Deletion of 4.1 kb of the IGDMR in mice results in almost abolished expression of the non-coding transcripts that are normally expressed maternally, and the activation of the maternal allele of the genes that are normally expressed paternally, *Dlk1, Rtl1*, and *Dio3* (Lin et al., 2003). Thus, the IGDMR is necessary for the maintenance of maternal silencing for the *Dio3* gene and for proper imprinted expression of other transcripts in the locus. We reasoned that if these regions were to harbor BN-SD polymorphisms, their effect on hippocampal allele-specific *Dio3* expression could be tested in a new reciprocal F1 rat cross.

## Materials and methods

### Animals

Animal procedures were approved by the Northwestern University Animal Care and Use Committee and conducted as described previously (Sittig et al., 2011a). Long Evans (LE) and BN females were crossed to BN or LE males, respectively, to generate F1 reciprocal offspring, LB and BL, at least 6 offspring/sex/group for fetal and adult analyses. For the fetal study, dams were sacrificed by decapitation on gestational day 21 (G21). Additional pregnant dams were allowed to give birth and rear litters. One or two males and one or two females from each litter were given behavioral tests between 10 and 20 weeks of age. Behavioral testing was staggered with age such that animals were tested in SI at about 10 weeks of age and OFT at 18 weeks of age. Adult animals were sacrificed by decapitation after a 2 week rest after behavioral testing, and their brains were used for allelic expression analyses. Additional brains from littermates were obtained from behaviorally naïve animals. Brain dissections of fetal and adult brains were performed as described (Sittig et al., 2011a).

### Behavioral testing

To test for anxiety behavior, we used two paradigms that have a well-established anxiety component, the open field test (OFT), and a social interaction paradigm. The OFT and social interaction tests (*N* = 5–10/strain/sex) were conducted on adult rats at >60 days of age as described previously (Sittig et al., 2011a, 2012), with >2 weeks' rest between tests. For OFT, TSE Videomot 2 software (version 5.75, Bad Homburg, Germany) was used to collect and analyze the data including total distance traveled and time spent in the outer and inner (50 cm diameter) regions. Rats were placed in a circular arena 82 cm in diameter for 10 min and allowed to freely explore while activity was tracked by the software (Nosek et al., 2008). The arena was lit with a brightness of 60 lux. For social interaction, adult rats were exposed to juvenile stimulus pups of 24–28 days of age and the interaction was video-recorded for 4 min. Adults were individually habituated in a testing cage for 5 min before beginning the first trial. A trained observer scored olfactory investigation of the adult toward the pup, defined as seconds spent in direct contact.

### *In silico* mapping and genomic analysis

We mapped the location of rat *Dio3* by using the basic local alignment sequence tool (BLAST) available from the National Center for Biotechnology Information (NCBI). *Dio3* has been mapped to Chr 6 random: 1,588,184–1,590,044 in the rat genomic assembly (Rat Genome Database, RGD_v3.4). To define the actual chromosomal location, we calculated the distance in base pairs between the *Dio3* transcript and the nearest correctly assembled gene, *Ppp2r5c*, the first non-imprinted gene located 3′ of the *Dlk1-Dio3* locus.

A rat homolog to the mouse IGDMR regulatory region was determined by homology to the 4.15 kb sequence which was deleted in the mouse (Lin et al., 2003). Polymorphic rates between rat strains were determined using SNPlotyper at the Rat Genome Database (http://snplotyper.mcw.edu/). To determine % polymorphic rates to the outbred SD strain we used the phylogenetic neighbors, SS/JrHsd and SR/JrHsd, and reported a range. A similar approach was used with LE/Han and Le/Stm for the LE rat. The BN strain used was BN/SSNHsd. The phylogenetic relationships among laboratory rat strains were presented previously (Saar et al., 2008). Transcription factor binding sites were predicted using Transcription Element Search System (www.cbil.upenn.edu/cgi-bin/tess/).

### *Dio3* pyrosequencing PCR and pyrosequencing

Pyrosequencing is a reliable method for validating allele-specific expression (Deveale et al., 2012). Methods for *Dio3* analyses including RNA extraction, reverse transcription, and PCR and pyrosequencing (*N* = 5–10/strain/sex/developmental stage/brain region) are identical to those described (Sittig et al., 2011a). Total RNA extraction was performed using Trizol reagent (Life Technologies, Gaithersburg, MD, USA) according to the manufacturer's protocol. Genomic DNA was removed using the TURBO DNA-*free* kit (Applied Biosystems, Foster City, CA, USA). DNased RNA (1 ug) was reverse transcribed using the TaqMan Reverse Transcription kit (Applied Biosystems, Branchburg, NJ, USA). Primers for PCR and pyrosequencing were reported previously (Sittig et al., 2011b).

### Sequencing of genomic regions

DNA extraction was performed with standard procedures as described (Sittig et al., 2012). PCR primers were designed using NCBI's Primer Design program. Sequencing primers were designed using the Saccharomyces Genome Database Web Primer program (http://www.yeastgenome.org/cgi-bin/web-primer). PCR and sequencing primers for *Dio3* promoter, as well as the initial sequencing of the rat IGDMR, are described in Table 1. The IGDMR was divided into five ~1kb sections (I–V) for sequencing purposes. PCR reactions (*N* = 3/strain) were verified in size (bp) by agarose gel electrophoresis and sequenced by ACGT, Inc. (Wheeling, IL).

**Table 1:** **PCR and sequencing primers for IGDMR and *Dio3* promoter**.

**Region**	**Section (5′–3′)**	**Sequence 5′–3′**	**bp product**
IGDMR	I	F: CCTCTAGGCTTTACTCGTGGGGG	1066
		R: AGGCGCTTGGCTCCTACCGT	
	I-sequencing	F: GTAGGTAGTACACCATCC	–
		R: AGTGTACCACGTAGCTGT	
	II	F: CGCTTCGTGGCGCAAACACAT	1000
		R: CGCGGCATTGTGAACCGTGG	
	II-sequencing	F: CGTGTACTATGTATGGTA	–
		R: CGTTTGTCTGGAGTACAC	
	III	F: TTGGGCCAGTCCCAGGGCT	960
		R: GACTCCTCTAGGATCCCCGGAGC	
	III-sequencing	F: TACTGTGCACAAGGACTG	–
		R: TTATGGGTCTTCCTTTTC	
	IV	F: CCTCCTCCCTAGGTTGCCCTTAGTA	1030
		R: TTGGCCAAGTGAGAGACATCTGCAA	
	IV-sequencing	F: ATCCTCATCTTCAAATAC	–
		R: AACAAGTAGGTTCAGGTG	
	V	F: TAGGAATGAGAGAGCCCTACCCCA	1059
		R: GCAAGCCTGCATGTATGTGCAGAG	
	V-sequencing	F: ACACCTGAACCTACTTGTT	–
		R: AGCTAGCTTGTTTGCAGG	
*Dio3* promoter	–452 to *Dio3*	F: CGCGGGAAGCGAACCGGAG	681
		R: GGTCAGGATGACGACGGCGC	
	Sequencing	F: TGCGGCCGCCGGGGAACTCA	–
		R: AGGAAATGCTTGCGGATG	

### Statistics

Data were first analyzed by Two-Way ANOVA using strain, sex, developmental stage, and brain region as factors where applicable. Data were pooled and re-analyzed by *t*-test if there was no interaction and no effect of either strain or sex in the initial analysis, or to test-specific hypotheses. Data are males and females combined unless otherwise noted. Significance indicated on figures represents the result of Bonferroni corrected *t*-tests.

## Results

### Mapping the *Dlk1-Dio3* locus in the rat

**Figure 1:**
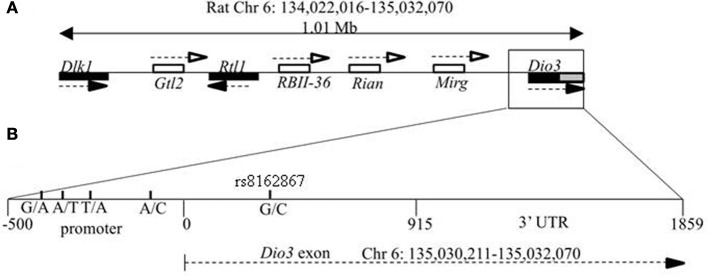
**Genomic location of the *Dlk1-Dio3* imprinted locus and the deiodinase-III (Dio3) gene. (A)** Relative position of the paternally expressed genes *Dlk1, Rtl1*, and *Dio3* (filled boxes), and maternally expressed non-coding transcripts *Gtl2*, RBII-36 C/D snoRNAs, *Rian*, and *Mirg* (open boxes). *Dio3* is located at the distal end of the imprinted locus and usually exhibits preferential paternal expression. *Dio3* contains a single exon (black) and 3′ untranslated region (gray). **(B)** Specific subregion which was sequenced for polymorphisms between BN vs. LE and SD strains. Four polymorphisms were found within the high-GC promoter region (−500 to 0) proximal to the *Dio3* start site (0). A synonymous G/C SNP in the *Dio3* exon (342) allows determination of paternal/maternal allelic expression. Chromosomal bp location of the *Dio3* transcript is given below. Location of genes and non-coding transcripts are not to scale.

The coding sequence and relative position of rat *Dio3* at the distal end of the *Dlk1-Dio3* locus is known, but the location of *Dio3* is currently reported as Chr 6 random: 1,588,184–1,590,044 region in the rat genomic assembly (Rat Genome Database, RGD_v3.4). Here, we mapped the 1859 bp *Dio3* coding exon and 3′UTR to rat Chr 6: 135,030,211–135,032,070. Figure 1 shows a schematic map of the rat *Dlk1-Dio3* imprinted locus, with its 3′ boundary designated as the 3′ end of *Dio3*. *Dlk1* has been mapped previously, so the *Dlk1-Dio3* imprinted region spans 1.01 Mb on Rat Chr 6: 134,022,016–135,032,070.

Based on our finding that hippocampal *Dio3* allelic expression differ between reciprocal crosses SB and BS and exhibited a maternal rather than paternal preference of allelic expression in the SD-mothered (SB) reciprocal cross, we aimed to identify genetic polymorphisms that explain this expression pattern. Several polymorphisms within the *Dlk1-Dio3* locus are not candidates for explaining region-specific allelic expression differences. First, a polymorphism between BN and SD strains has previously been identified at bp 342 of the *Dio3* exon (rs8162867, Figure 1). In our previous reports we have used this synonymous polymorphism to follow allelic expression patterns of *Dio3* in reciprocal BN-SD crosses (Sittig et al., 2011a,b). This G/C rs8162867 SNP could affect transcription differently across brain regions only if a hippocampal-specific transcription factor were to bind there. We searched for such binding sites across this SNP for both the “C” and “G” alleles, and found one hit for the “G” allele, the negative glucocorticoid response element (R01813). If glucocorticoid receptor does bind to the G allele, this may explain the hippocampal-specific repression of the paternal *Dio3* expression in SB and LB animals only if hippocampal-specific accessory factors allow binding in the hippocampus but not frontal cortex.

**Figure 2:**
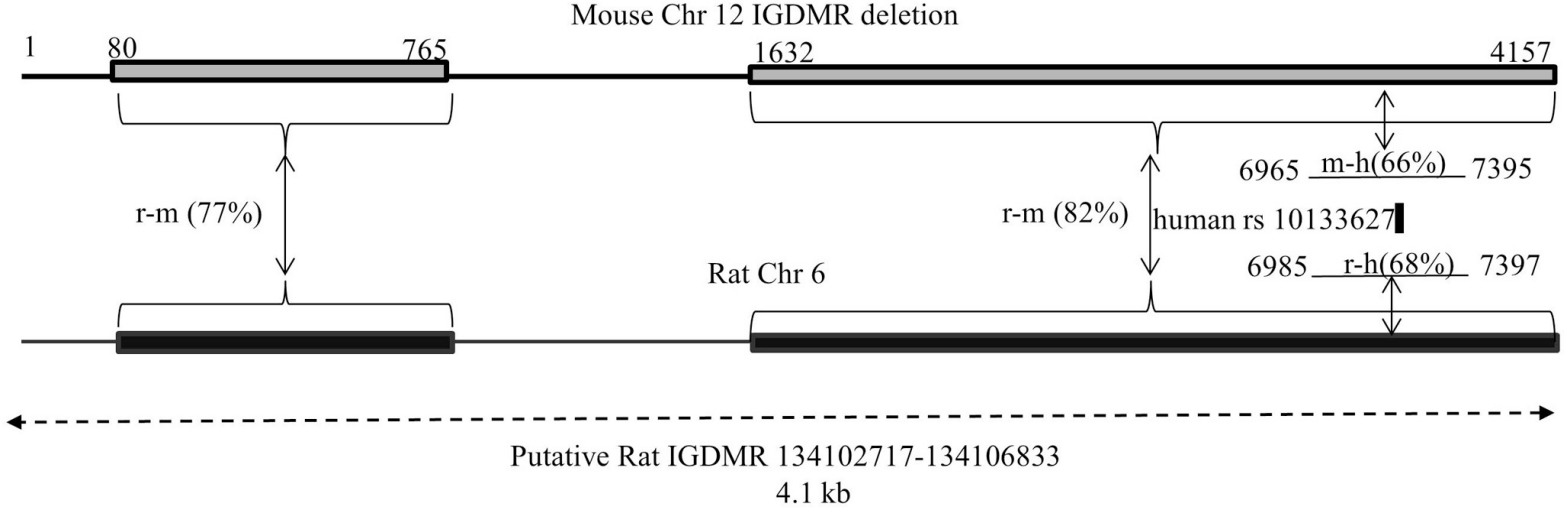
**Mapping the *Dlk1-Dio3* IGDMR on chromosome 6 in the rat.** Lin et al. created a targeted deletion of 4.15 kb of the mouse IGDMR [Chr 12, AJ320506 nt 79883–84039, top (Lin et al., 2003)]. When transmitted maternally, the deletion affects imprinted expression of the genes in the *Dlk1-Dio3* locus. We compared the deletion sequence to rat Chr 6 by NCBI's BLAST, defining the resulting homologous regions (rat-mouse 77 and 82%, respectively) and the intervening sequence on rat Chr 6 as the putative rat IGDMR. In the human, a maternally transmitted familial heterozygous 8.5 kb deletion encompassing the human IGDMR (Chr 14, NT_026437 82270449–82279006) manifested in uniparental disomy-like phenotypes (Kagami et al., 2010). A portion of this human deletion (6965–7397) including the human SNP rs10133627 maps within the mouse IGDMR and the rat sequence we identify as such. Numbering of mouse and human regions reflects bp number within each deletion. Preliminary DNA sequencing revealed no polymorphisms between BN and SD rats in the putative rat IGDMR region.

A second potentially regulatory region is the IGDMR. It was demonstrated that the maternal IGDMR is necessary for maintenance of maternally expressed non-coding transcript expression, and for the repression of the maternal copy of paternally expressed genes in the locus, including *Dio3* (Lin et al., 2003). Genetic differences at the IGDMR would likely affect imprinted expression at the entire locus, and for *Dio3*, would affect it across tissues rather than producing a hippocampal-specific effect. We deemed it unlikely that the IGDMR harbors a mutation in the SD or BN strain for this reason and because neither strain displays any of the severe developmental phenotypes associated with IGDMR mutations and/or disruption of imprinting at the *Dlk1-Dio3* locus (Lin et al., 2003, 2007; Kagami et al., 2010). Nevertheless, we mapped and sequenced the rat IGDMR as identified based on homology to the mouse IGMDR (PCR and sequencing primers in Table 1). This 4.1 kb region showed no polymorphisms between SD and BN strains. A schematic of mapping the putative rat IGDMR (Chr 6: 134,022,016–135,032,070) is given in Figure 2.

### Four novel SNPs within the proximal *Dio3* promoter

**Figure 3:**
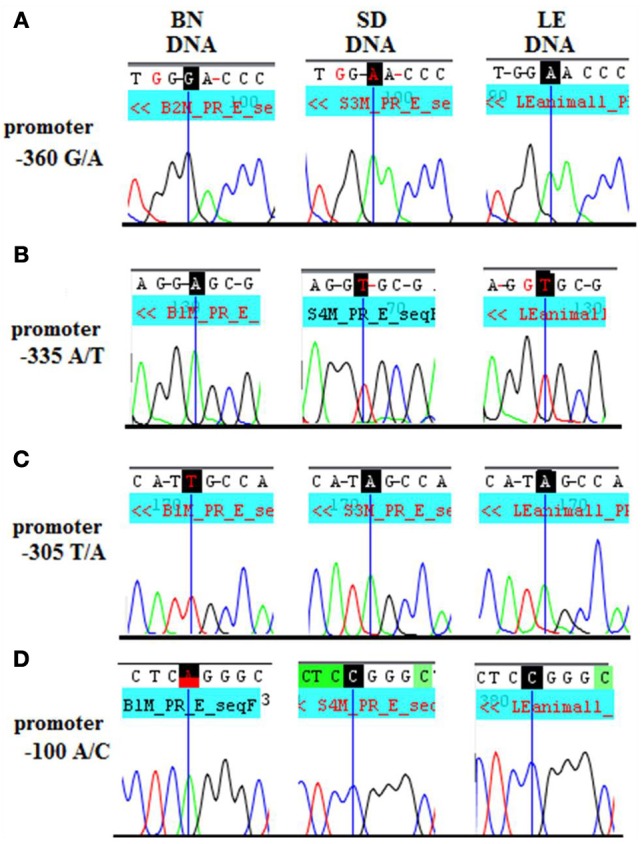
**Standard sequencing traces of four novel polymorphisms within the *Dio3* promoter region and their location relative to the *Dio3* start site. (A)** G/A at −360 bp upstream of *Dio3*; **(B)** A/T at −335 bp; **(C)** T/A at −305 bp; and A/C at −100 bp. BN genotype is given first, followed by SD and LE. BN genotype differs from the matching SD and LE genotype at each polymorphic site and **(D)** A/C at −100bp.

A strong candidate region that could influence *Dio3* allelic expression patterns region-specifically is the *Dio3* promoter. For example, if there were a hippocampal-specific transcription factor binding site or alternative promoter, a polymorphism could affect hippocampal transcription factor binding asymmetrically in the two reciprocal crosses. At least four differentially sized *Dio3* transcripts in rat brain (Tu et al., 1999) suggest the possibility of a brain region-specific promoter(s). Promoter activity *in vitro* has been shown for 1.8 kb upstream from *Dio3* in mouse and human with the strongest promoter activity occurring ~450 bp upstream from the transcription start site (Hernandez and St. Germain, 2003). This putative promoter region (1.8 kb–0 bp upstream of the *Dio3* start site) exhibits 92% identity between mouse and rat, and the 450 bp most proximal to *Dio3* exhibits 96% identity and 74% GC content. Four BN/SD polymorphisms were identified within the most proximal subregion of the putative rat *Dio3* promoter, −360, −335, −305, and −100 bp upstream from the *Dio3* coding sequence (Figures 3A–D).

### LE recapitulates the SD genotype in the *Dio3* exon and its promoter

To determine whether there is a genetic underpinning for brain region-specific variations in *Dio3* allelic expression and its consequences, we evaluated *Dio3* allelic expression in a new reciprocal cross. If SD-BN genetic polymorphisms within the *Dlk1-Dio3* imprinted region are causative toward variant *Dio3* allelic expression observed in SB vs. BS rats, then we should observe a similar pattern of *Dio3* expression in a different reciprocal cross where the SD has been replaced with a genetically similar strain. Since BN is phylogenetically divergent from other laboratory rat strains (Saar et al., 2008), we utilized the BN in a new cross. For the other strain we wanted to maintain the SD haplotype in the *Dio3* promoter but otherwise introduce genetic polymorphisms. We genotyped several candidate strains at several regions (data not shown), including the LE, which is phylogenetically close to the SD. The adult male LE rat also has similar peripheral TSH and T4 levels as the SD (Pekary et al., 1984), indicating there are not major genetic differences in thyroid function between these strains. The LE rat matched the SD genotype at all regions and all polymorphic sites identified in the *Dio3* region (Figures 3A–D), yet exhibits 29–31% polymorphic rate to SD-like strains. Thus, the LE is a genetically divergent strain in which many loci would be polymorphic with the SD, but the *Dio3* promoter region and exon are the same.

### LB-BL reciprocal crosses replicate allelic *Dio3* expression patterns in hippocampus with differences in anxiety-related behavior

**Figure 4:**
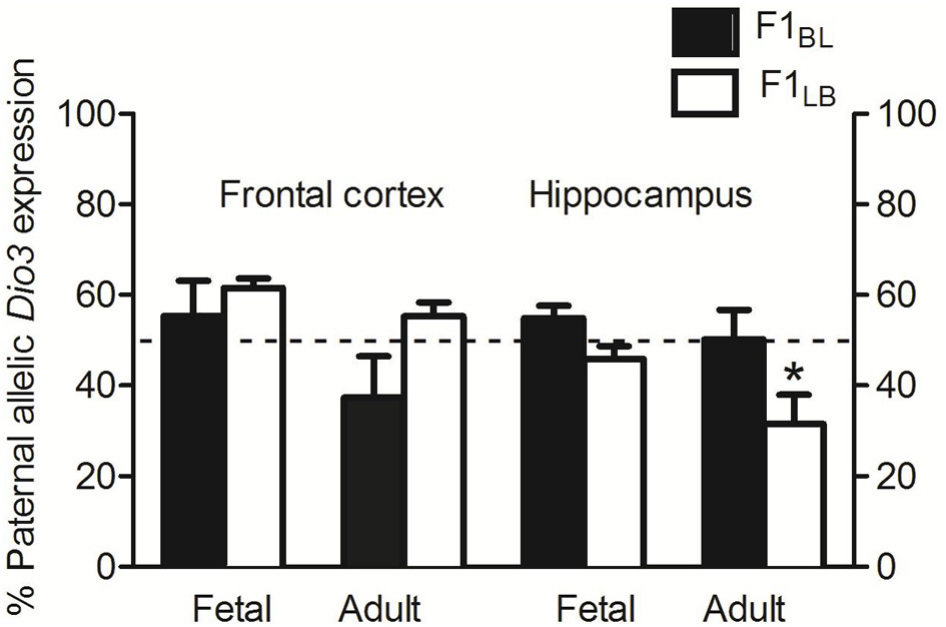
**Hippocampal-specific maternal *Dio3* allelic expression in LB reciprocally crossed offspring with anxiety-related behavior.** LB animals exhibit differential *Dio3* allelic expression in hippocampus only, while BL offspring show no significant deviations from the expected allelic expression pattern of *Dio3* in either brain region. Data represent means of individual animals' *Dio3* allelic expression values determined by quantitative pyrosequencing of cDNAs from each brain region. Results from animals that underwent behavioral testing did not differ from naïve animals. *N* = 12–14 individual fetal frontal cortex/strain, and *N* = 12–14 individual fetal hippocampal regions/strain. *N* = 12–13 individual adult frontal cortex/strain, and *N* = 13–15 individual adult hippocampal regions/strain. Data were first analyzed by Two-Way ANOVA using strain, sex, developmental stage, and brain region as factors where applicable. Data were pooled and re-analyzed by *t*-test if there was no interaction and no effect of either strain or sex in the initial analysis, or to test specific hypotheses. Data are males and females combined unless otherwise noted. ^*^p < 0.05 vs. frontal cortex, Bonferroni corrected.

As expected, BL animals exhibited slightly paternal to biallelic expression in the fetal frontal cortex and hippocampus and biallelic expression in the adult hippocampus, confirming the results seen previously in the BS reciprocal cross (Figure 4). BL *Dio3* expression in adult frontal cortex averaged slightly less than 50% paternal, but this appears to be an artifact of higher variability. In contrast, LB frontal cortex and hippocampus showed divergent *Dio3* expression patterns (Figure 4). There was a non-significant trend toward an interaction between strain and brain region for *Dio3* allelic expression in fetal frontal cortex and hippocampus [*F*_(1, 49)_ = 2.6, *p* = 0.11], and adults showed a significant interaction [*F*_(1, 49)_ = 7.8, *p* < 0.01]. Specifically, LB adults exhibited preferentially maternal *Dio3* expression in the hippocampus but preferentially paternal *Dio3* expression in the frontal cortex (Figure 4). These findings recapitulate the unique maternal preference for *Dio3* allelic expression that was reported in SB animals only in the hippocampus (Sittig et al., 2011a).

**Figure 5:**
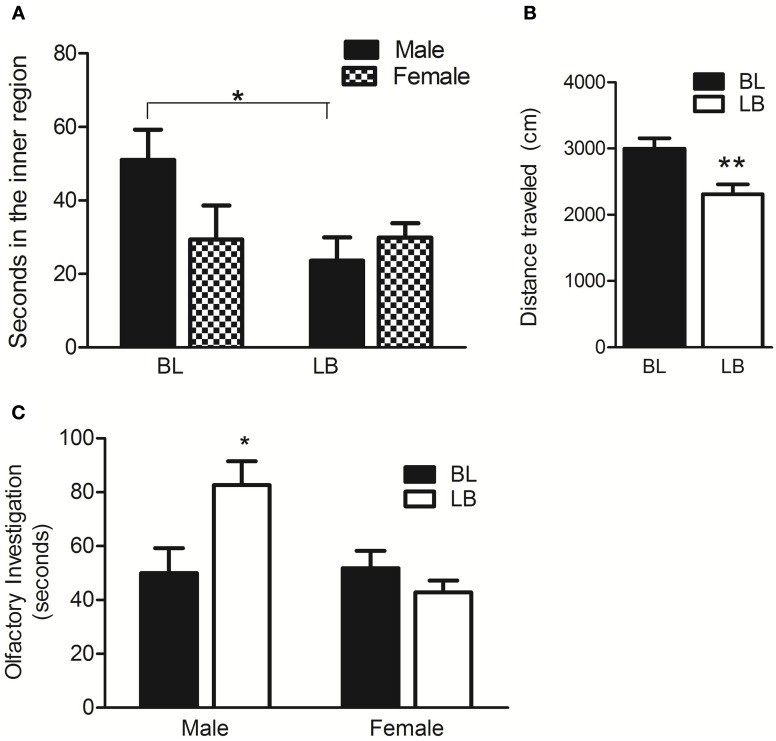
**Behavioral phenotypes indicate that LB animals have heightened anxiety in a novel environment but not during social interaction with a juvenile. (A)** LB males show heightened anxiety by spending less time in the inner region of the open field test than BL males. *N* = 9 LB males, *N* = 6 LB females; *N* = 5 BL males, *N* = 6 BL females. **(B)** LB animals traveled less total distance in the open field test compared to BL animals. **(C)** LB males showed a higher level of olfactory investigation of a juvenile pup than BL animals. *N* = 10 LB males, *N* = 10 LB females; *N* = 6 BL males, *N* = 6 BL females. Data were first analyzed by Two-Way ANOVA using strain and sex as factors. Data were pooled and re-analyzed by *t*-test if there was no interaction and no effect of either strain or sex in the initial analysis, or to test specific hypotheses. Data are males and females combined unless otherwise noted. ^*^p < 0.05, ^**^p < 0.01, Bonferroni corrected.

Previously, SB animals showing maternal hippocampal *Dio3* expression exhibited anxiety-like behaviors compared to the reciprocal BS cross. We therefore tested BL and LB animals on a well-established measure for anxiety, the OFT. LB males and females spent more time in the outer, anxiety-reducing portion of the circular OFT arena compared to BL animals [*t*_(25)_ = 2.6, *p* < 0.05, data not shown], suggesting a higher level of anxiety. Figure 5A shows that LB males only spent less time in the inner, anxiogenic region of the arena [*t*_(12)_ = 2.6, *p* < 0.05; hypothesis testing due to trends of strain and sex in the ANOVA], suggesting that the anxiety phenotype was more pronounced in the LB males than in LB females. LB animals also traveled less distance than BL [*F*_(1, 23)_ = 8.7, *p* < 0.01] (Figure 5B), a phenotype which is known to occur concomitant with other anxiety-like behaviors in rats (Naqvi et al., 2012; Rosenfeld and Weller, 2012). Finally, we asked whether the social anxiety exhibited by SB was also present in LB of the current study. We found that LB males in fact displayed more social investigation than BL [interaction of sex and strain: *F*_(1, 28)_ = 7.1, *p* < 0.05] (Figure 5C). This finding indicates that it is very unlikely that the LB animals' hypoactivity in the OFT was due to a motor deficit.

## Discussion

The experiment presented here is an approximated parent-of-origin specific congenic experiment which confirms the effect of the SD genotype at the *Dio3* promoter when inherited maternally. Our original reciprocal cross parental strains, BN and SD, exhibit a polymorphic rate of ~62%. Since the SD-LE exhibit ~31% polymorphic rate, there are significant genetic differences between the SB-BS vs. LB-BL reciprocal crosses. Furthermore, the BN-LE polymorphic rate is 51–52%, more than 10% less than BN-SD. Here we have taken advantage of the fact that the polymorphisms in SB-BS vs. LB-BL reciprocal crosses do not differ in the *Dio3* promoter region but do differ elsewhere. There are several potential mechanisms of the hippocampal-specific *Dio3* gene expression, including differential expression of alternative *Dio3* transcripts, differential use of alternative promoters, and polymorphisms in cis- or trans-acting transcription factor binding sites or transcription factors themselves. Whichever of these is true, the eventual elucidation of the correct mechanism may involve cis-polymorphisms close to the *Dio3* gene itself. The current work reveals the specific polymorphisms at the *Dio3* promoter region which could be mechanistically involved in the strain- and brain region- specific regulation of gene expression. Mechanistic proof of the *Dio3* promoter polymorphisms' effects could be carried out in primary cultures from cortex and hippocampus of our reciprocal crosses.

Our data is consistent with the idea that *Dio3* is one of many genes which by small variations in its expression, affects brain circuits that control complex, multigenic behaviors including anxiety-like behavior. LB animals were less active than BL in the OFT, which could be interpreted as anxiety-related behavioral inhibition in a novel situation. However, hypoactivity alone is not likely to account for the fact that LB males spent less time in the center of the OFT since LB females showed less total activity but spent the same time in the center of the maze as BL females. In addition, LB males interacted more with juvenile stimulus pup in social interaction, a behavior that is highly dependent on intact mobility. Additional behavioral assays would be required to firmly associate *Dio3* expression with anxiety-like behavior, but our current data indicate the proof-of-concept that LB rats behaved differently from BL rats on a classic, stable measure of anxiety: time spent in the periphery of the open field.

In this report, we first mapped and sequenced genomic regions that likely contribute to an unexpected and novel finding, aberrant allelic expression of the thyroid hormone metabolizing gene *Dio3* in the hippocampus of one reciprocal rat cross. We confirmed this novel pattern of expression using a different reciprocal cross harboring the same genetic differences in the promoter region of *Dio3*, indicating that parent-of-origin *Dio3* expression in the hippocampus is of genetic origin, and confirmed the co-occurrence of anxiety-like behavior. We cannot exclude the possibility that other SNPs within the *Dlk1-Dio3* locus (e.g., in the ncRNA transcripts found there) and/or outside the locus are important for the findings about allelic expression patterns reported herein. Polymorphisms across the genome and not only in and around *Dio3* are likely to contribute to the behavioral differences between reciprocal crosses, but these effects are at a minimum in the current study, since strains being compared are two reciprocal F1 crosses and are therefore genetically identical. In humans, parent-of-origin specific genetic effects modulating brain function are a possibility that is beginning to undergo exploration. Should human orthologs for polymorphisms within *Dio3* be found, they could be important in explaining differences in neuropsychiatric vulnerability and cognitive functioning. In the future, polymorphisms that lead to brain region-specific allelic gene expression, as well as the genetic × epigenetic interactive mechanisms of this specificity, will need to be elucidated in both rodent models and humans.

### Conflict of interest statement

The authors declare that the research was conducted in the absence of any commercial or financial relationships that could be construed as a potential conflict of interest.
